# MAGE-A11 is a potential prognostic biomarker and immunotherapeutic target in gastric cancer

**DOI:** 10.18632/aging.205368

**Published:** 2024-01-04

**Authors:** Zhi-Wen Wang, Qi-Ying Yu, Meng-Jiao Xu, Chuan-Yi Zhou, Jia-Peng Li, Xing-Hua Liao

**Affiliations:** 1 Institute of Biology and Medicine, College of Life Sciences and Health, Wuhan University of Science and Technology, Wuhan 430081, Hubei, P.R. China; 2 Central Laboratory, Tumor Hospital Affiliated to Nantong University, Nantong 226361, Jiangsu, P.R. China; 3 Key Laboratory of Chronic Noncommunicable Diseases, Yueyang Vocational Technical College, Yueyang 414006, Hunan, P.R. China; 4 Zhaoyuan Linglong Central Health Center, Zhaoyuan 265400, Shandong, P.R. China; 5 College of Science, Wuhan University of Science and Technology, Wuhan 430081, Hubei, P.R. China; 6 Yueyang People’s Hospital, Yueyang Hospital Affiliated to Hunan Normal University Neoplasm Ward 1, Yueyang 414000, Hunan, P.R. China

**Keywords:** MAGE-A11, gastric cancer, prognostic value, immune infiltration

## Abstract

Gastric cancer poses a serious threat to human health and affects the digestive system. The lack of early symptoms and a dearth of effective identification methods make diagnosis difficult, with many patients only receiving a definitive diagnosis at a malignant stage, causing them to miss out on optimal therapeutic interventions. Melanoma-associated antigen-A (MAGE-A) is part of the MAGE family and falls under the cancer/testis antigen (CTA) category. The MAGE-A subfamily plays a significant role in tumorigenesis, proliferation and migration. The expression, prognosis and function of MAGE-A family members in GC, however, remain unclear. Our research and screening have shown that MAGE-A11 was highly expressed in GC tissues and was associated with poor patient prognosis. Additionally, MAGE-A11 functioned as an independent prognostic factor in GC through Cox regression analysis, and its expression showed significant correlation with both tumour immune cell infiltration and responsiveness to immunotherapy. Our data further indicated that MAGE-A11 regulated GC cell proliferation and migration. Subsequently, our findings propose that MAGE-A11 may operate as a prognostic factor, having potential as an immunotherapy target for GC.

## INTRODUCTION

Immunotherapy is one of the most promising cancer treatments and is often used in the advanced stages of most tumors [1]. One of the prerequisites for tumor immunotherapy is the recognition of tumor antigens, which will then trigger subsequent specific immune responses to control and removal tumors. Tumor antigens are divided into tumor-specific antigens and tumor-associated antigens. Cancer/testis antigens (CTAs) are a large family of tumor-associated antigens and only their expression is restricted to germline and tumor cells [2]. Among the CTAs families, MAGE-A, NY-ESO-1, LAGE1, and TTK are considered potential therapeutic targets due to their *in vivo* immunogenicity and specific expression patterns and have entered early stages of clinical trials [3–5].

Among cancer/testis antigens members, MAGE-A subfamily was the first identified gene family, which has also been deeply studied. MAGE-A (melanoma associated antigens-a) belongs to MAGE superfamily and its members include MAGE-A1-MAGE-A12 whose encoding gene are all located on X chromosome [6, 7]. Aberrant expression of MAGE-A family members has been identified in not just melanoma but also breast, bladder, lung, ovarian, and hepatic cancers [8–12]. Based on these findings and their expression characteristics, members of the MAGE-A family are considered good potential immunotherapeutic targets. However, the expression, function and prognosis of MAGE-A family members in GC are poorly understood. In this study, we analyzed the expression differences among family members in normal and tumor tissues, as well as their expression and prognostic value under different clinical characteristics. Immune cell infiltration and immunotherapy analyses were also performed which will provide theoretical basis for investigating the role of MAGE-A family members in immunotherapy of GC.

## MATERIALS AND METHODS

### Expression and survival analysis of MAGE-A protein family genes

We downloaded expression data and corresponding clinical data from the UCSC Xena database and the TCGA database. The differential expression was then analyzed using the R packages limma and ggplot2. When drawing the survival curve, we utilized the online database Kaplan-Meier plotter (http://kmplot.com/analysis/index.php?p=background) and also utilized the R packages to process the corresponding clinical data and expression data of TCGA.

### Screening for differentially expressed genes

The samples were divided into high expression group and low expression group of MAGE-A11 according to the mean expression level of MAGE-A11. The data were then processed with the R packages limma, ggplot2 and pheatmap to screen for differentially expressed genes and presented the results in the form of a heatmap [13, 14].

### Analysis of independent prognostic factors

MAGE-A11 expression, age, gender, Grade and Stage were included in Cox regression analyses. Subsequent time-dependent ROC curves were used to judge the accuracy and specificity of univariate and multivariate Cox regression analysis results. Finally, we constructed a nomogram. The nomogram contains 8 factors which are: age, gender, M, N, T, Stage, Grade and MAGE-A11 expression.

### Functional enrichment analysis

To study the biological function of differentially expressed genes, Gene Ontology enrichment analysis was performed using the R package “clusterProfiler. FDR ≤ 0.05 was considered statistically significant [15–17].

### Tumor immune cell infiltration analysis

The CIBERSORT algorithm was used to complete the analysis of immune-infiltrating cells in each sample [18, 19].

### EdU assay

Cells were cultured on a 14mm cell culture coverslide at 37° C with 5% CO2 overnight according to the EdU kit instructions (Thermo Fisher Scientific, USA). When the cells reached 70-80% confluence, the medium was eliminated, washed twice with PBS, and subsequently incubated in complete medium containing 50 mM EdU for 2 hours. Finally, reagents were added sequentially according to the instructions.

### Cellular immunofluorescence

Ki67 as well as E-cadherin (Abclonal, China) were diluted according to the manufacturer’s instructions and incubated on cell slides overnight. Secondary antibodies were added and incubated for one hour, followed by observation and photography.

### Transwell assay

Cell migration ability was tested using the Transwell assay under different treatment groups. Technical abbreviations are explained on first use. Cells undergoing logarithmic growth were treated. The cell suspension was adjusted to a density of 5×10^5^/mL and 100 μL was added into the Transwell chamber. Complete medium was added to 24-well plate, and incubation carried out for 24 hours. The results were observed after crystal violet staining.

### Construction of xenograft tumor model

Three-week male nude mice purchased from Beijing Huafukang Experimental Animal Co., Ltd. 2 x10^7^ cells from the knockdown group and the control group were injected subcutaneously into the nude mouse. After 18 days of incubation, animal imaging was performed.

### Statistical analysis

All data analyses in this study were performed with R software version 4.1.0. The Wilcoxon test was used to compare significant differences between the two groups of data. Kaplan-Meier analysis was used to examine survival curves. Construction of univariate and multivariate Cox analyses was based on proportional hazards models. The T-test was used to calculate whether there was a statistical difference between the two groups. p-values less than 0.05 were considered statistically significant.

### Availability of supporting data

The data generated during this study are included in this article and its supplementary information files are available from the corresponding author on reasonable request.

### Consent for publication

All authors have read this manuscript and approved for submission.

## RESULTS

### Expression of MAGE-A family in gastric cancer

**Figure 1:**
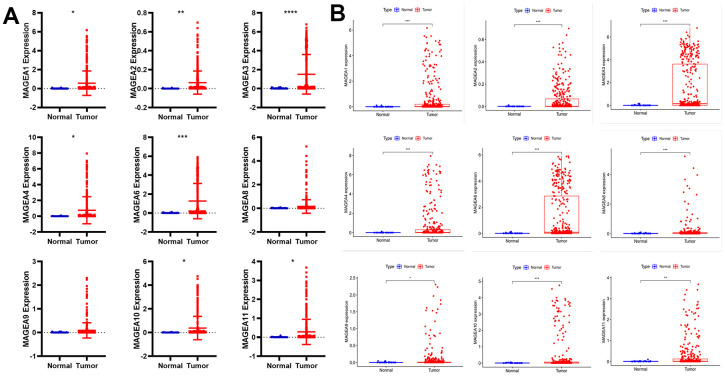
**Differential expression analysis of MAGE-A family members between tumor and normal tissues.** (**A**) Pan-cancer data analysis of MAGEA family members expression in gastric cancer. (**B**) Expression of MAGEA family members was analyzed from TCGA gastric cancer expression data.

First, we downloaded sequencing data for 33 cancers from the UCSC Xena database (http://xena.ucsc.edu/). We then extracted the expression data of MAGE-A family members and plotted them as scatter plots (Figure 1A). These results showed that MAGE-A1, MAGE-A2, MAGE-A3, MAGE-A4, MAGE-A6, MAGE-A10 and MAGE-A11 were significantly highly expressed in cancer tissues, while the remaining family members had no significant difference in expression. Finally, we also downloaded the data of the TCGA database and performed differential expression analysis of MAGE-A family members in cancer and adjacent tissues (Figure 1B). We found that MAGE-A all family members were significantly highly expressed in cancer tissues.

### Analysis of the prognostic role of the MAGE-A family

**Figure 2:**
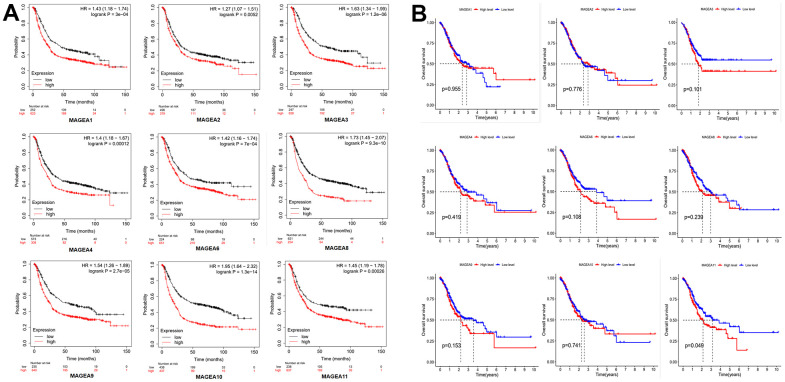
**Prognostic role of MAGE-A family members.** (**A**) Survival curve from the website Kaplan-Meier plotter. (**B**) Survival curves constructed by TCGA clinical and expression data.

To verify the prognostic role of MAGE-A family members in GC. We first applied the online database Kaplan-Meier plotter (http://kmplot.com/analysis/index.php?p=background) to plot survival curves of members of the MAGE-A family. The results were shown in Figure 2A, the high expression group of family members showed poor prognosis of the patients. We then validated these results by applying Kaplan-Meier analysis to the expression data and clinical information downloaded from TCGA. We found that only the survival curve for MAGE-A11 was statistically significant (Figure 2B). Moreover, high expression of MAGE-A11 also represented a poor prognosis. Together with these findings, we selected MAGE-A11 as a follow-up study target.

### Expression of MAGE-A11 in different clinical features

**Figure 3:**
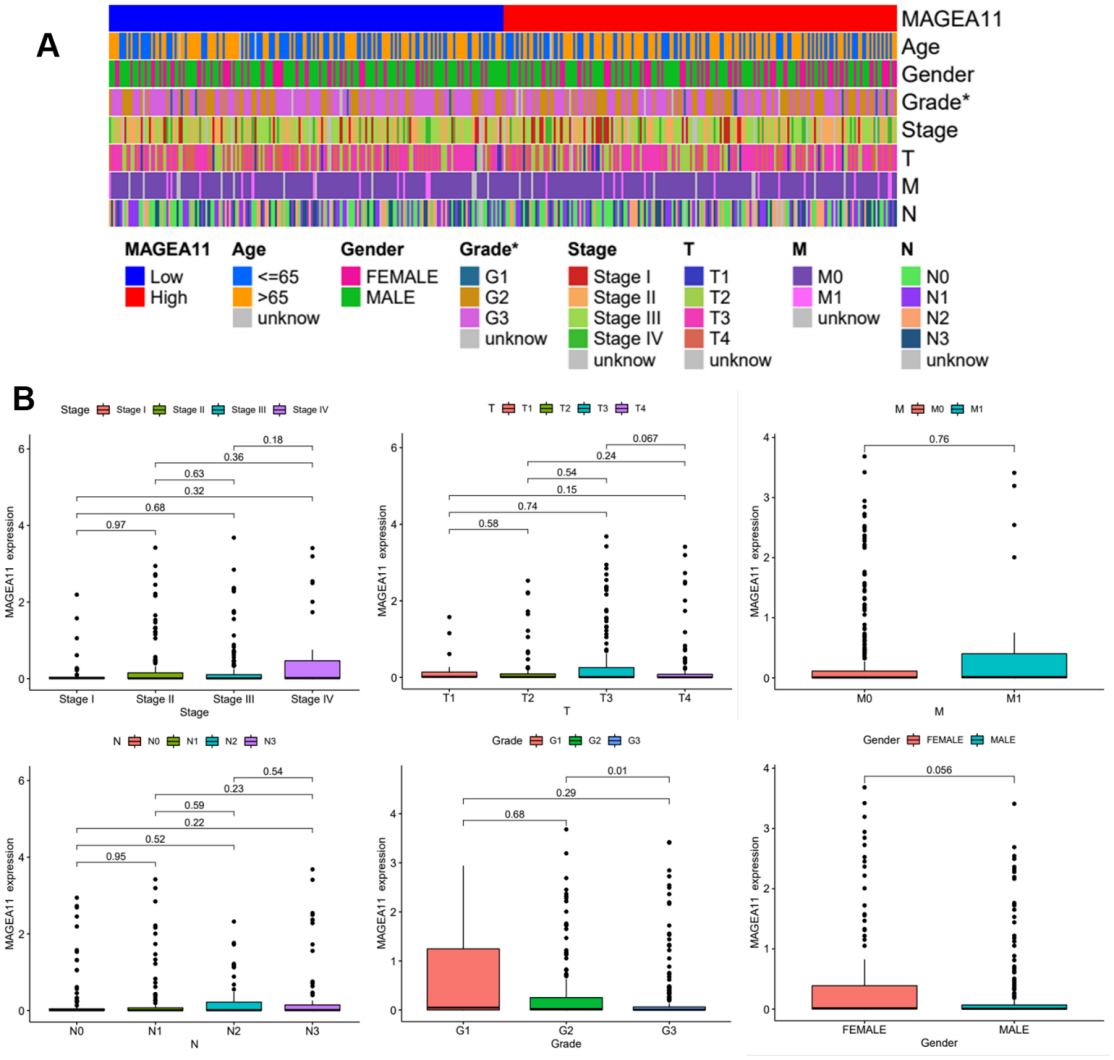
**Expression of MAGE-A11 in different clinical features.** (**A**) Clinical heat map of MAGE-A11 expression. (**B**) Box plot of MAGE-A11 expression under different clinical characteristics.

We plotted MAGE-A11 clinical heatmap using the limma and ComplexHeatmap R packages (Figure 3A). The findings indicated that there was a statistically significant difference in the expression of MAGE-A11 among Grade, but not in relation to M, N, T, gender, age, or tumor stage. To further determine the details of MAGE-A11 expression in the Grade classification, box plots were ploted based on clinical data. As shown in Figure 3B, MAGE-A11 was significantly differentially expressed in G2 and G3 while the other groups were not statistically different. These expression differences suggest that it is more accurate to employ Grade staging when using MAGE-A11 as a therapeutic target and prognostic factor.

### MAGE-A11 is an independent prognostic factor for gastric cancer

**Figure 4:**
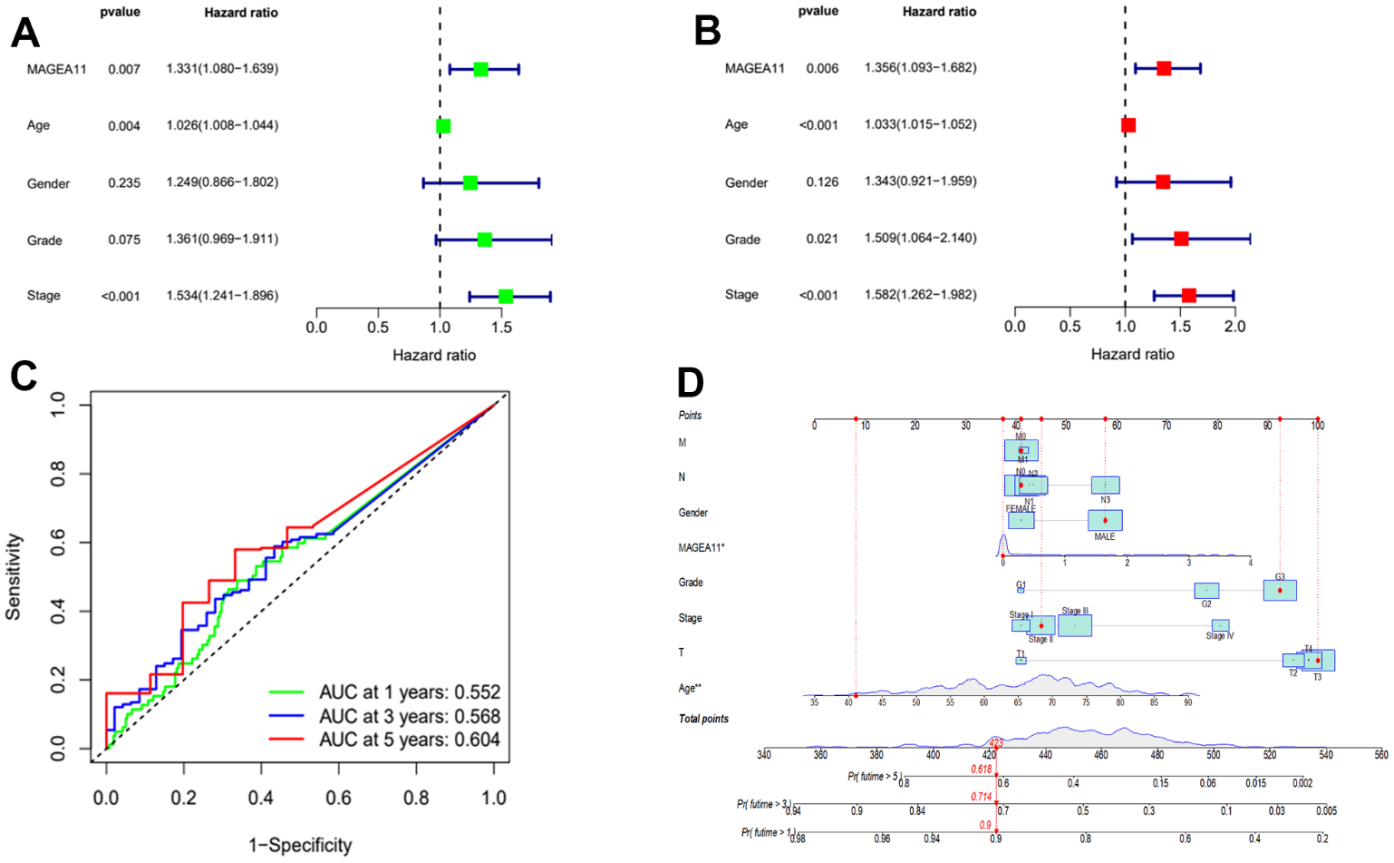
**MAGE-A11 is an independent prognostic factor.** (**A**) Univariate Cox regression analysis of the expression and clinical characteristics of MAGE-A11. (**B**) Multivariate Cox regression analysis of the expression and clinical characteristics of MAGE-A11. (**C**) Time-dependent ROC curves. (**D**) The nomogram is applied by adding up the points identified on the points scale for each variable. ROC: receiver operating characteristic curve. AUC: area under the curve.

After determining the expression of MAGE-A11 under different clinical features, we continued to investigate its prognostic role in GC. We incorporated MAGE-A11 expression and clinical information into univariate and multivariate Cox regression analysis and found that MAGE-A11 expression, age and Stage were independent predictors of GC (Figure 4A, 4B and Table 1). We then applied time-dependent ROC curves to verify the accuracy of the above findings. The results were shown in Figure 4C, The AUC values all exceeded 0.5. These values indicated the accuracy and specificity of the results of the Cox regression analysis described above. We constructed a nomogram for its prognostic value (Figure 4D).

**Table 1:** Results of the MAGE-A11 expression and clinical characteristics in the univariate and multivariate Cox regression analysis.

**ID**	**Univariate Cox regression analysis**					**Multivariate Cox regression analysis**			
	**HR**	**HR.95L**	**HR.95H**	**P-value**		**HR**	**HR.95L**	**HR.95H**	**P-value**
**MAGE-A11**	1.330649	1.080051	1.639391	**0.00729**		1.355726	1.092814	1.681891	**0.005659**
**Age**	1.025793	1.008122	1.043774	**0.004074**		1.033345	1.014846	1.052183	**0.000373**
**Gender**	1.249052	0.865632	1.802303	**0.234564**		1.343008	0.920908	1.958579	**0.125534**
**Grade**	1.360868	0.969129	1.910957	**0.075253**		1.508955	1.064031	2.139923	**0.02099**
**Stage**	1.533532	1.240617	1.895606	**7.70E-05**		1.581805	1.262205	1.98233	**6.83E-05**

Bold words mean P-value is less than 0.05; HR, hazard ratio; L, low; H, high.

### Identification of differentially expressed genes and functional enrichment analysis

**Figure 5:**
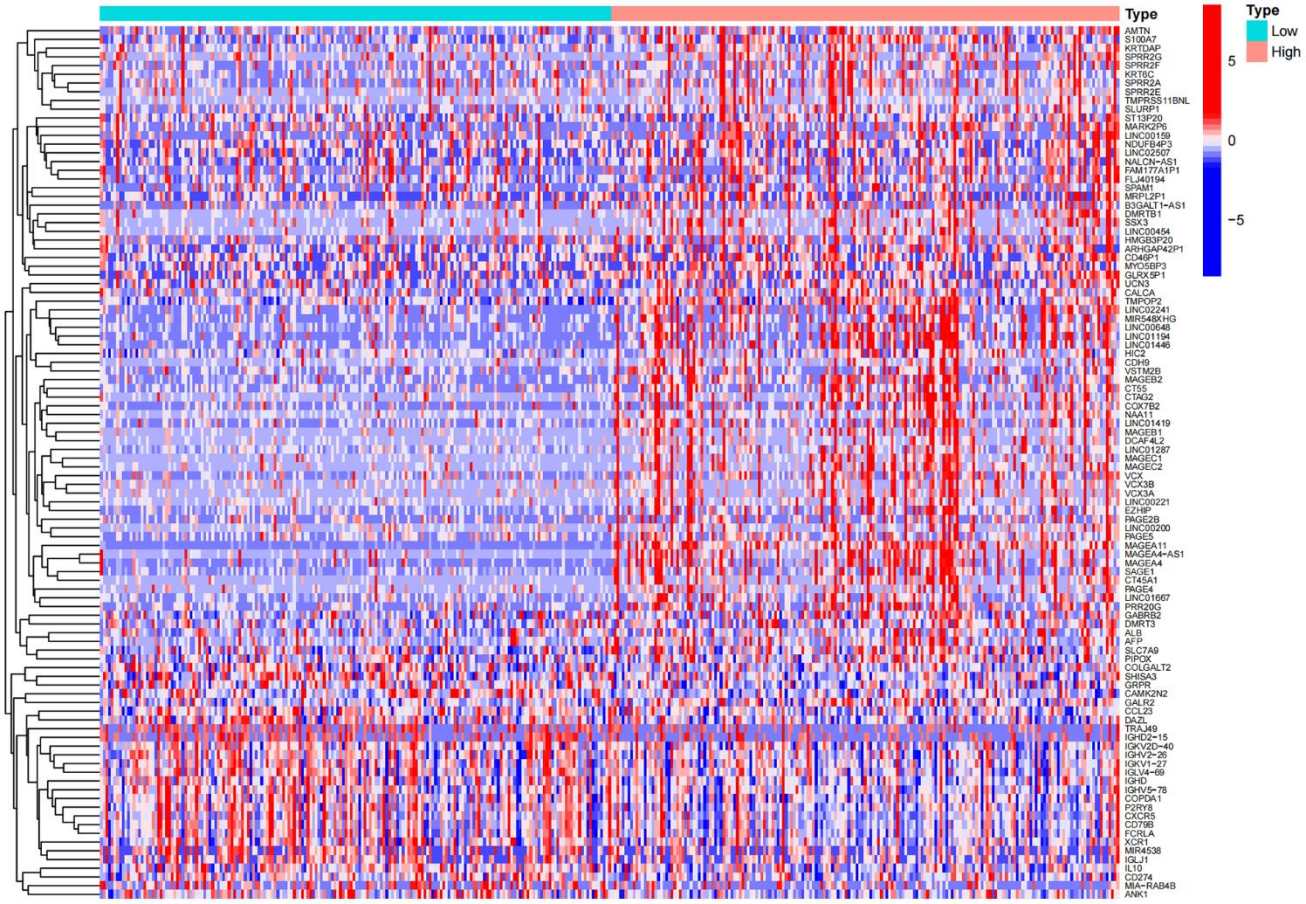
Heat map of differential genes in the high and low expression groups of MAGE-A11.

**Figure 6:**
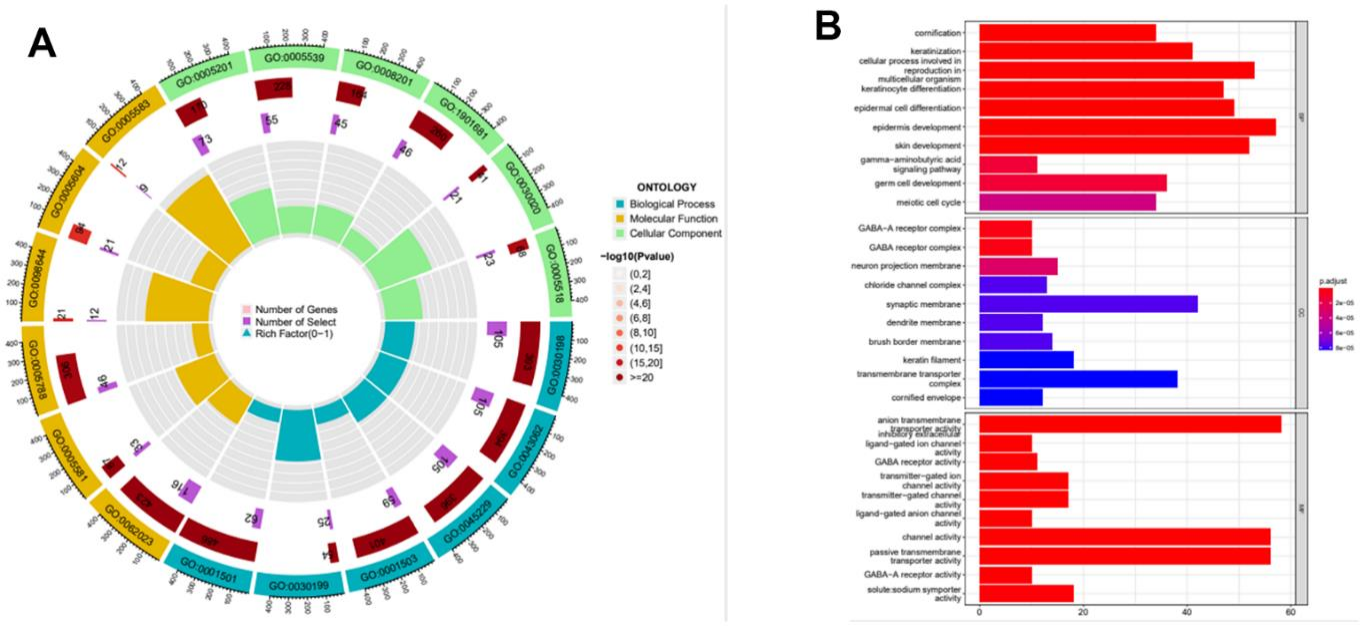
**Functional enrichment analysis of the expression profile of MAGE-A11.** (**A**) Functional enrichment analysis circle diagram. (**B**) Functional enrichment analysis histogram.

According to the expression level of MAGE-A11, we divided the expression data of GC into MAGE-A11 high and low expression groups. And based on this as a basis for the screening of differential genes. We applied the limma and pheatmap R packages to process the data and plot the top 50 differentially expressed genes into a heatmap (Figure 5). Finally, we found 2075 differentially expressed genes, of which 27 were down-regulated and 2,048 genes were up-regulated. Then we carried out biological function analysis of these differential genes. The results of GO enrichment analysis showed that MAGE-A11 was involved in epidermis development and other functions (Figure 6A, 6B).

### The relationship between the expression of MAGE-A11 and the tumor microenvironment

**Figure 7:**
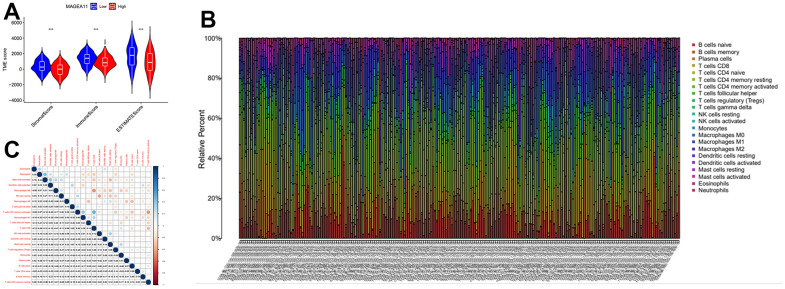
**The relationship between MAGE-A11 expression and the tumour microenvironment.** (**A**) Stromalscore, Immunescore and ESTIMATE score differences between MAGE-A11 high and low expression groups. (**B**) Proportion of tumor-infiltrating immune cells in each sample. (**C**) Relationship between tumor-infiltrating cells.

**Figure 8:**
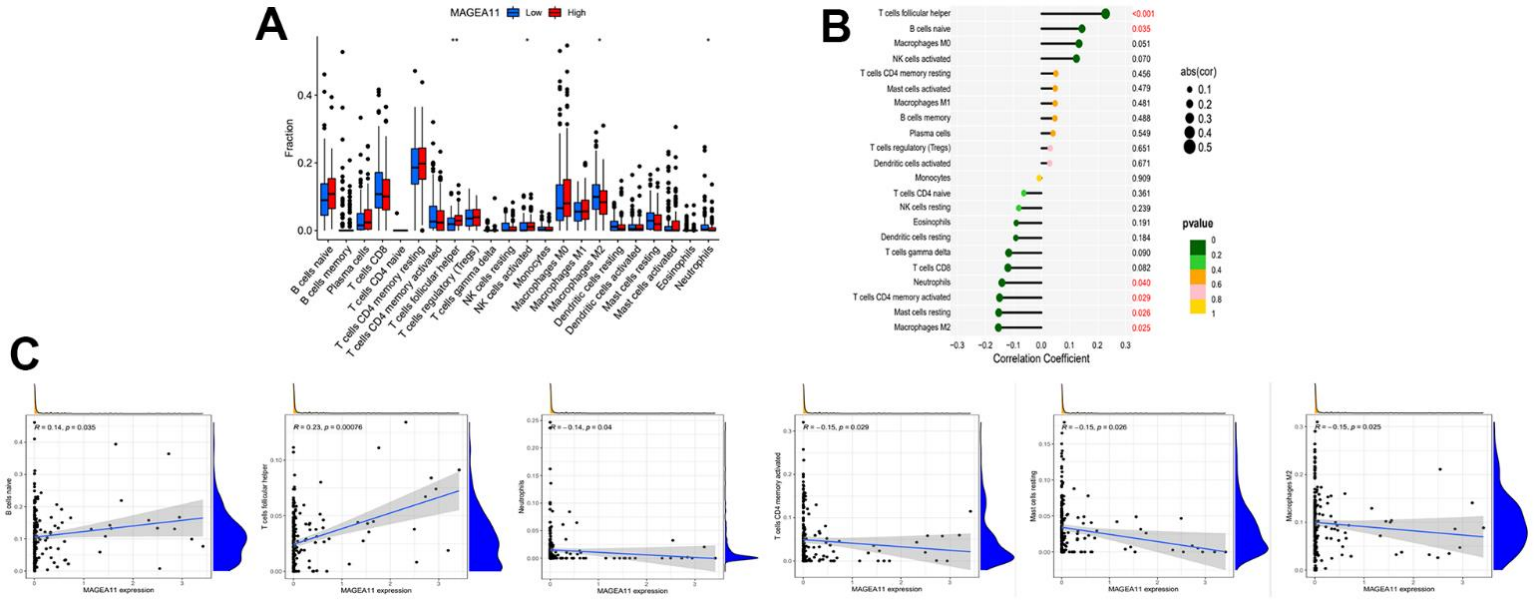
**Relationship between tumor-infiltrating cells and MAGE-A11 expression.** (**A**) Box plots of the proportions of tumor-infiltrating cell types in tumor tissues with low (blue) or high (red) MAGE-A11 expression. (**B**) Map of the proportional relationship between MAGE-A11 expression and tumor-infiltrating cells. (**C**) Scatter plots showing Pearson’s correlation between the proportions of the 6 most significant and MAGE-A11 expression.

**Figure 9:**
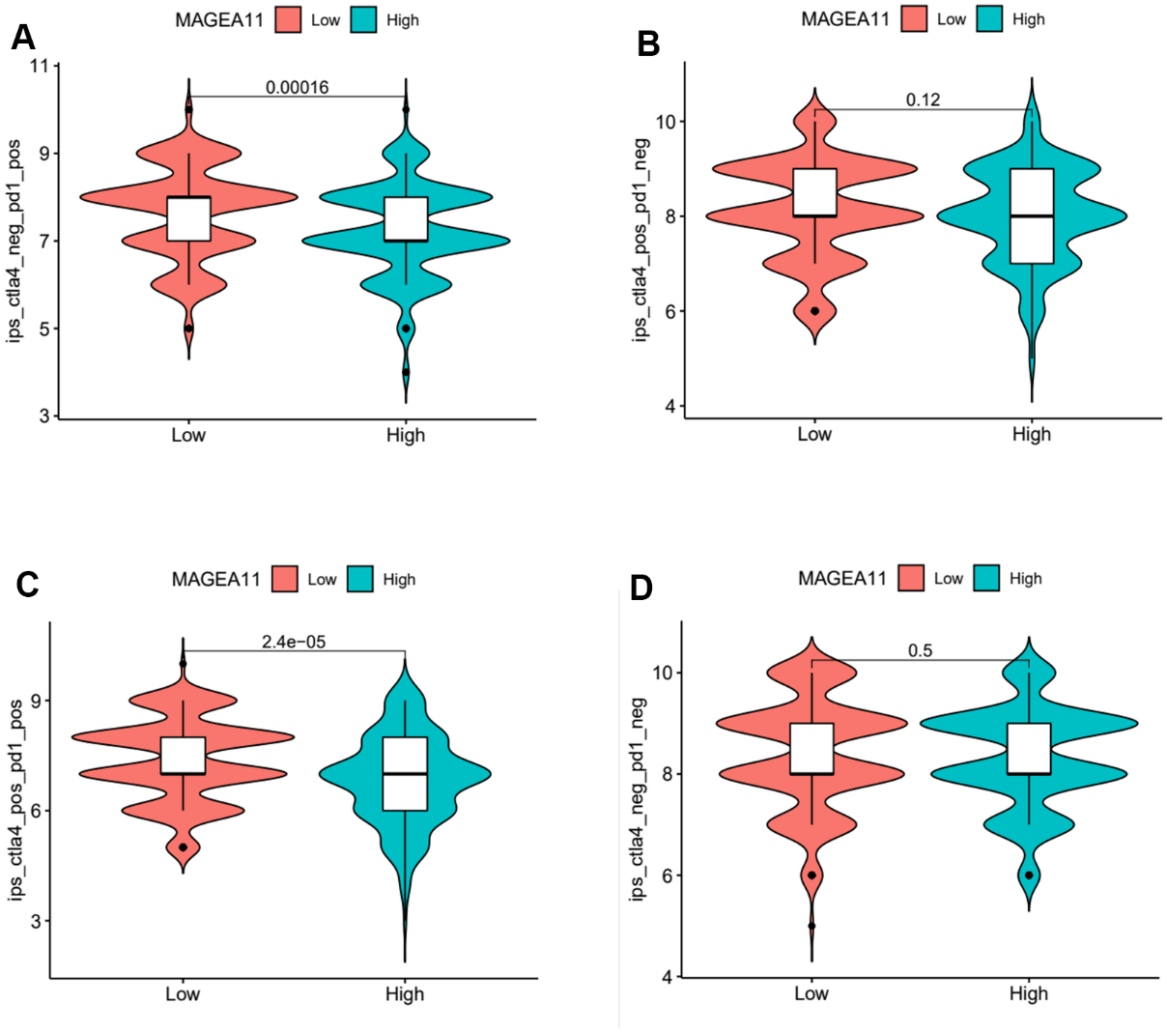
**Violin plot of MAGE-A11 expression and sensitivity to immunotherapy.** (**A**) Anti-PD1 immunotherapy. (**B**) Anti-CTLA4 immunotherapy. (**C**) Anti-PD1 and CTLA4 immunotherapy. (**D**) Non-immunotherapy.

Next, we investigated the relationship between MAGE-A11 expression and the tumour microenvironment. Firstly, the samples were grouped based on their high and low MAGE-A11 expression levels, and subsequent analyses of Stromalscore, Immunescore, and ESTIMATE scores were conducted to determine the differences between the respective expression groups. As shown in Figure 7A, there were significant differences in the analysis results, and the score of the MAGE-A11 low expression group was significantly higher than that of the high expression group. This implied a strong relationship between the expression of MAGE-A11 and the tumor microenvironment. In order to explain the relationship between the expression of MAGE-A11 and the tumor microenvironment in more detail. We applied the CIBERSORT algorithm to calculate the proportions of 22 types of infiltrating cells in each tumor sample and plotted them as bar graphs (Figure 7B). Correlations between infiltrating cells were also analyzed and plotted as a heat map (Figure 7C). Then we analyzed the difference of immune infiltrating cells (Figure 8A) and the Pearson’s relationship between each infiltrating cell and MAGE-A11 expression (Figure 8B, 8C). The results of the analysis showed that a total of six infiltrating cells were associated with the expression of MAGE-A11. Among them, T cells follicular helper and B cells naive cells were positively correlated with its expression, and the rest were negatively correlated. Finally, we analyzed the relationship between the expression of MAGE-A11 and immunotherapy. As shown in Figure 9, in general, the MAGE-A11 low expression group had better effect when receiving immunotherapy than the high expression group, and the effect was the most obvious when receiving combined immunotherapy against PD1 and CTLA4 (Figure 9C), followed by PD1 or CTLA4 treatment alone (Figure 9A, 9B). There was no significant difference between the two groups without treatment (Figure 9D). This suggested that when MAGE-A11 was selected as a therapeutic target, the combination of immune checkpoint therapy may achieve better results.

### MAGE-A11 regulates the proliferation and migration of GC cells

**Figure 10:**
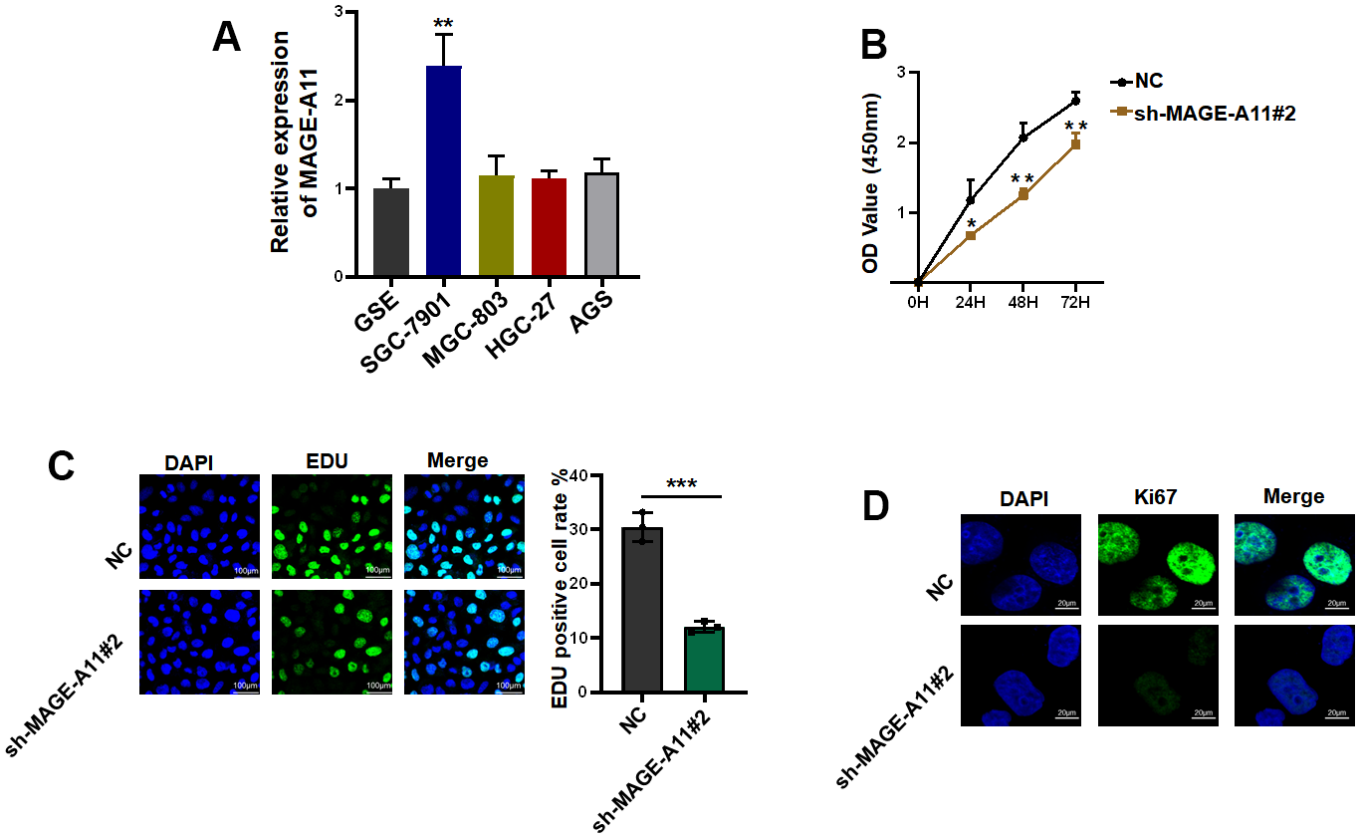
**MAGE-A11 has the ability to regulate the proliferation of tumor cells.** (**A**) The expression of MAGE-A11 was detected by QPCR in tumor cells and gastric epithelial cells. (**B**) CCK-8 assay was used to detect the effect of MAGE-A11 knockdown on cell viability. (**C**) EdU assay was used to detect the effect of MAGE-A11 knockdown on cell proliferation. (**D**) The expression of Ki67 was detected by cellular immunofluorescence.

**Figure 11:**
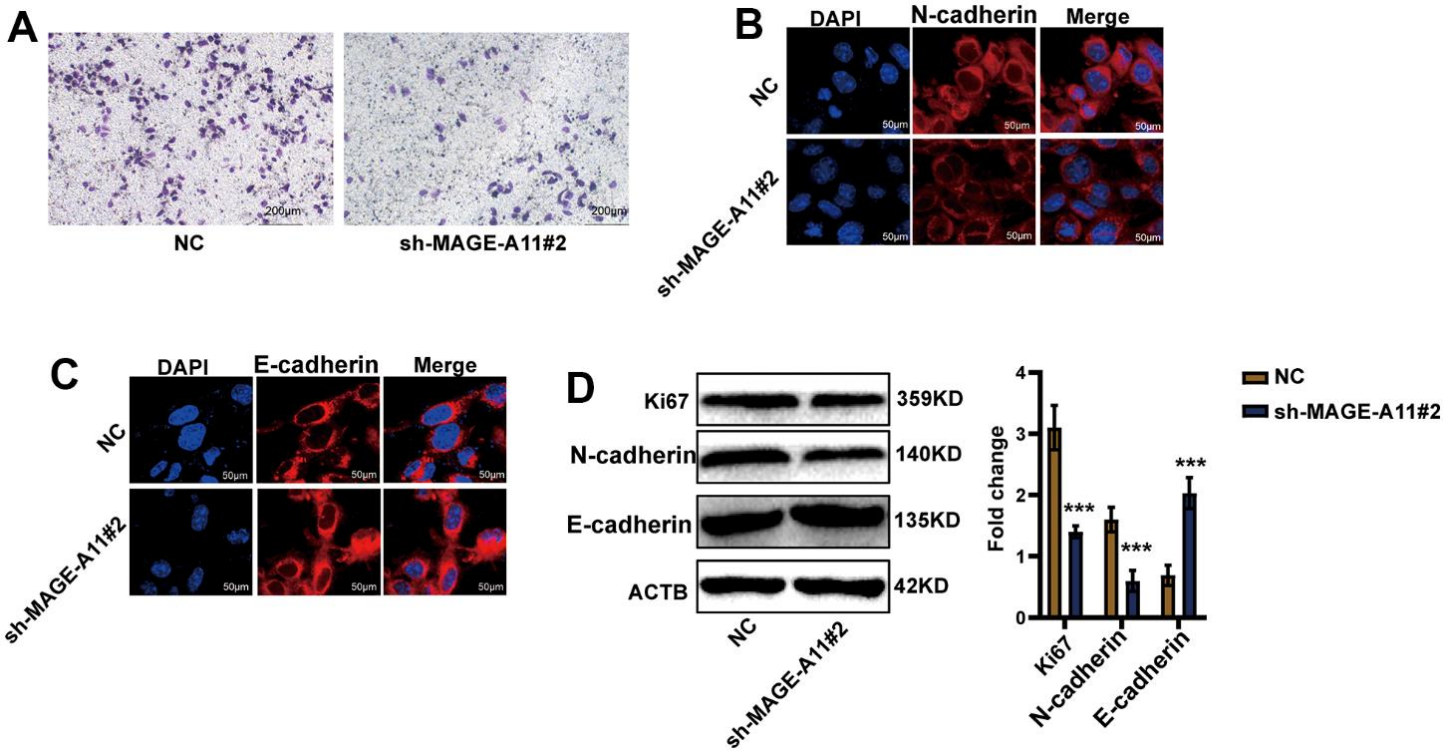
**AGE-A11 has the ability to regulate tumor cell migration.** (**A**) Transwell assay was used to detect the effect of MAGE-A11 knockout on cell migration ability. (**B**) Detection of N-cadherin by cellular immunofluorescence. (**C**) Detection of E-cadherin by cellular immunofluorescence. (**D**) The changes of cell proliferation and migration protein markers were detected by western blot.

Firstly, we detected the expression of MAGE-A11 in GC cells and GC epithelial cell line by qPCR. As shown in Figure 10A, the expression level of MAGE-A11 in SGC-7901 cells was significantly higher than that of other GC cell lines. In order to study the biological function of MAGE-A11, a stable expression cell line of MAGE-A11 was constructed. Subsequently, we tested the effect of its knockout on cell proliferation by different methods respectively. CCK-8 cell activity assay detected that MAGE-A11 knockout reduced the activity of GC cells by about 24% (Figure 10B). The EdU assay detected the DNA synthesis ability and rate of cells during proliferation, and the positive rate of EdU decreased significantly in the knockdown group (18.4%) (Figure 10C). We also detected the expression of the Ki67. In accordance with the above results, the expression of Ki67 decreased significantly in the knockdown group (Figure 10D). Transwell assay and cellular immunofluorescence assay of N-cadherin and E-cadherin showed that MAGE-A11 also has the ability to regulate cell migration (Figure 11A–11C). Finally, we found that the protein markers of cell proliferation and migration changed significantly after the knockout of MAGE-A11 (Figure 11D).

**Figure 12:**
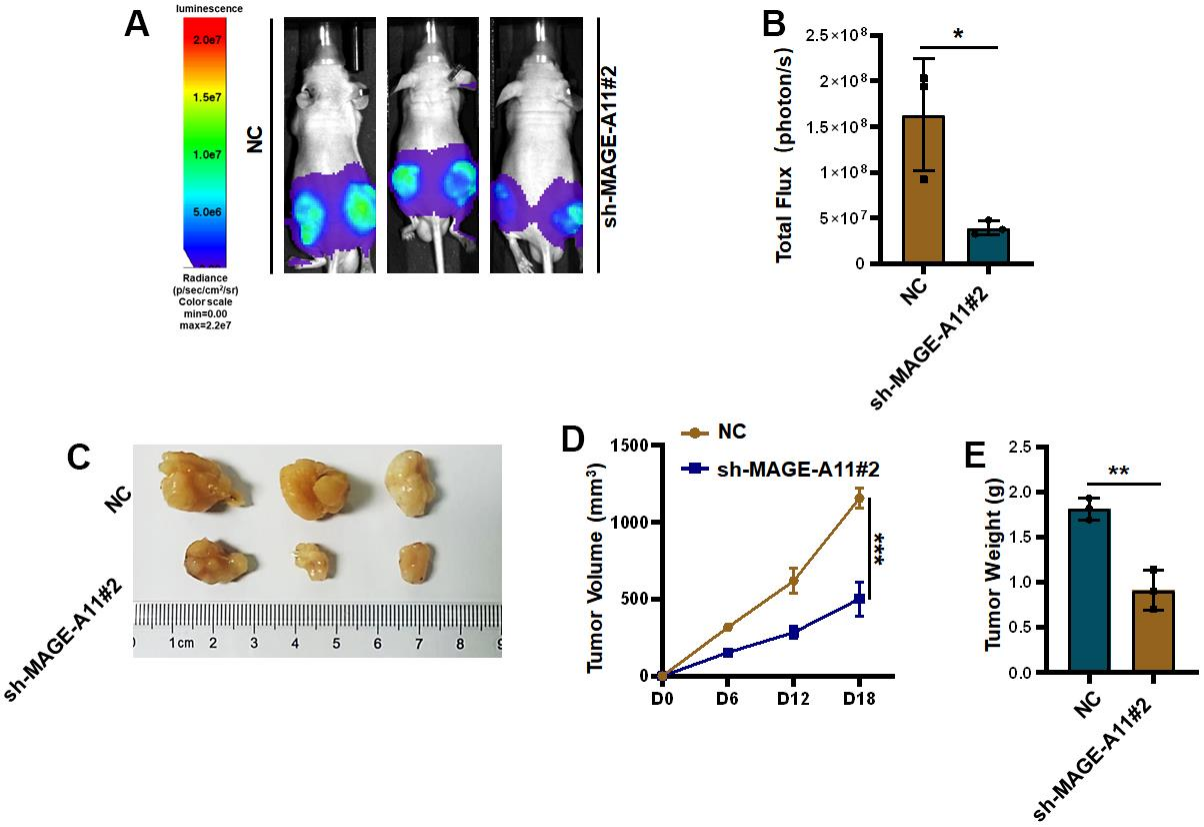
**Knockout of MAGE-A11 affects tumor growth *in vivo*.** (**A**) Animal imaging image. (**B**) Fluorescence value of animal imaging. (**C**) Image of tumor in experimental group and control group. (**D**) Tumor volume curve (**E**) tumor weight.

Finally, the effect of MAGE-A11 on tumor *in vivo* was detected, so we established a tumor model in nude mice. Tumor volume records were measured at 6, 12, and 18 day. On the eighteenth day after subcutaneous injection of cells from the experimental group and the control group, the tumorous mice were subjected to animal imaging, and then the mice were killed, stripped of the tumors, photographed and weighed. As shown in Figure 12A, 12B, fluorescence values of tumors in the knockdown group decreased significantly. In Figure 12C, the picture of the dissected mouse tumor showed that the overall volume of the knockdown group was smaller. Tumor volume curves showed a reduction of 654mm^3^ in tumor volume and an average reduction of 0.9g in the knockdown group at day 18 of tumor inoculation (Figure 12D, 12E).

## DISCUSSION

GC remains one of the most prevalent cancers [20]. Due to the asymptomatic fact in the earlier stage, the five-year survival rate for patients after diagnosis remains poor and despite significant advances in antineoplastic treatment [21]. There is therefore an urgent need for an antineoplastic method that will improve patient survival rate and treatment outcomes.

Immunotherapy shows promise as an effective treatment [22]. The MAGE-A subfamily, which is the most extensively researched group among the cancer/testis antigens, is viewed as a possible target for immunotherapy and is therefore incorporated into clinical studies. Tumour vaccines that contain MAGE- A3 have been developed and analysed in clinical trials for melanoma and non-small cell lung cancer patients [23, 24]. Meanwhile, MAGE-A1, MAGE-A3, MAGE-A4, and MAGE-A10 have been identified as targets for TCR-T cell immunotherapy [25].

Members of the MAGE-A subfamily are also involved in the process of tumour development. Zhao and his colleagues discovered that MAGE-A1 can impact the proliferation and migration of tumors in breast and ovarian cancers by influencing the NOTCH signaling pathway. This was achieved by decreasing the stability of NCID through affecting its ubiquitination modifications [26]. MAGE-A2 was highly expressed in cancers such as glioma, lung cancer and embryonal carcinoma and is closely associated with poor patient prognosis [27–29]. Hideki Uj and colleagues found that MAGE-A2 played a prognostic role in lung cancer and may promote tumor by regulating the p53 signaling pathway [28]. Studies have reported that MAGE-A3 was highly expressed in tumours such as gastric, bladder, prostate, colon and melanoma [30]. This suggested that MAGE-A3 played a vital role in tumourigenesis and progression. MAGE-A11 was highly expressed in esophageal squamous cell carcinoma, head and neck squamous cell carcinoma and retinoblastoma and was involved in tumour resistance, proliferation, migration and apoptosis [31–34]. However, this study provided the initial suggestion that MAGE-A11 was an autonomous prognostic aspect in GC, and its manifestation associated closely with the tumor immune microenvironment.

The study identified that MAGE-A11 had an autonomous prognostic effect and regulated tumour cell proliferation and migration. However, the research has some limitations that should be scrutinized for further study and exploration. For instance, the regulation of the molecular mechanisms of cell proliferation, migration and cellular immunity is attributed to MAGE- A11. It remains to be investigated whether MAGE-A11’s ability to regulate the tumor microenvironment will have an impact on the condition of tumor stem cells. Additionally, the question of whether MAGE-A11 exerts similar influence in other types of tumours merits further investigation.

Overall, the results of our study indicated that MAGE-A11 may be an independent prognostic factor for GC patients. Additionally, we observed that MAGE-A11 was capable of promoting tumour cell proliferation and migration. These findings suggested that MAGE-A11 could be a valuable target for therapeutic intervention in GC.
